# Identification of candidate genes harboring pathogenic variants in congenital heart disease and laterality defects in Chinese population

**DOI:** 10.3389/fgene.2025.1582718

**Published:** 2025-05-08

**Authors:** Jinxin Wang, Weicheng Chen, Xianghui Huang, Han Gao, Zhiyu Feng, Chaozhong Tan, Quannan Zhuang, Yuan Gao, Shaojie Min, Yuquan Lu, Feizhen Wu, Maoxiang Qian, Weili Yan, Wei Sheng, Guoying Huang

**Affiliations:** ^1^ Pediatric Heart Center, Children’s Hospital of Fudan University, Shanghai, China; ^2^ Shanghai Key Laboratory of Birth Defects, Shanghai, China; ^3^ Fujian Key Laboratory of Neonatal Diseases, Children’s Hospital of Fudan University at Xiamen (Xiamen Children’s Hospital), Fujian, China; ^4^ Research Unit of Early Intervention of Genetically Related Childhood Cardiovascular Diseases (2018RU002), Chinese Academy of Medical Sciences, Shanghai, China

**Keywords:** congenital heart disease, laterality defects, Chinese populations, candidate genes, pathogenic variants

## Abstract

**Objective:**

Congenital heart disease (CHD) is often accompanied by laterality defects (LD), giving rise to a severe and intricate form of congenital anomaly. The aim of this study was to explore the genetic etiology of CHD/LD in the Chinese population.

**Methods:**

We recruited 52 Chinese CHD family trios between January 2008 and August 2019, each comprising a CHD/LD proband and their healthy parents. Whole exome sequencing (WES) was carried out on peripheral blood samples from these trios. Candidate genes harboring pathogenic variants were determined through quality control of WES results and a screening approach based on variant rarity, deleteriousness, inheritance patterns, and gene function.

**Results:**

A total of two candidate genes and 46 CHD-related genes harboring LOF (loss-of-function) variants were identified. These included one *de novo* variants (in *DNAH2*), two compound heterozygous variants (in *DNAH2*), and one X-linked recessive variants (in *FLNA*). Significantly, cilia-related genes *DNAH2* had the highest frequencies of variants. Additionally, 26.1% (12/46) of CHD-related genes harboring LOF variants were significantly linked to cilia function.

**Conclusion:**

This research identified two novel candidate genes (*DNAH2*, and *FLNA*) for CHD/LD in the Chinese population, with *DNAH2* ciliary genes being the most frequently occurring among all candidate genes. The results offer critical insights into the genetic basis of CHD/LD in the Chinese population, which may have implications for genetic counseling and prenatal prevention.

## 1 Introduction

Congenital heart disease (CHD) affects about 1% of live births and is the most common cause of morbidity and death among congenital abnormalities (Liu et al., 2019; Zhang et al., 2021; Xiao et al., 2023). Laterality defects (LD), including heterotaxy and situs inversus totalis (SIT), are a class of congenital abnormalities, occurring in approximately 1 in 6,000 to 1 in 9,000 live births (Lin et al., 2014; Reller et al., 2008). These defects are characterized by abnormal positions of visceral structures. There is a significant association between LD and CHD, with a high percentage of patients with heterotaxy or SIT also presenting complex CHD. During embryonic development, the heart undergoes intricate bending from the symmetric heart tube within the mesodermal midline to form a four-chambered blood-pumping organ predominantly located in the left thoracic cavity, exhibiting significant asymmetry. Consequently, there is an undeniable association between LD and CHD, with 82.8% of heterotaxy patients and 26.6% of SIT patients presenting with complex CHD (Lin et al., 2014). Early identification and intervention are crucial for infants with combined LD and CHD to prevent adverse outcomes.

The etiological study of these conditions is crucial for genetic counselling and prenatal prevention. Non-heritable maternal factors, such as alcohol, drugs, or diabetes, have been associated with LD and CHD (van Veenendaal et al., 2016; Kuehl and Loffredo, 2002). Genetic variables, including chromosomal abnormalities, copy number variations (CNVs), and certain syndromes, are strongly linked to LD (Pierpont et al., 2018; Yi et al., 2022). Additionally, monogenic pathogenesis involving genes such as *ZIC3*, *GDF1*, *ACVR2B*, and *MMP21* contribute significantly to these disorders (Li et al., 2018; Gao et al., 2019; Kosaki et al., 1999; Qin et al., 2022). A distinct class of monogenic genes related to cilia, including *DNAAF1*, *DNAAF2*, *DNAAF3*, *DNAAF5*, *DNAH5*, *DNAH6*, and *DNAI* are conclusively associated with LD (Antony et al., 2021; Braun and Hildebrandt, 2017; Li et al., 2015). These genes play key roles in establishing the left-right asymmetric signaling pathway, affecting the development of CHD/LD (Pierpont et al., 2018; Reiter and Leroux, 2017). However, the causative genes can vary across different genetic backgrounds (Kingdom and Wright, 2022). The limited ability to generalize the findings mentioned above to diverse population backgrounds or the small sample sizes highlights the need for larger family trio studies in the Chinese population to identify genes harboring pathogenic variants. Our primary objective was to thoroughly investigate the genetic origins of CHD/LD within the Chinese population, aiming to uncover key insights into their genetic etiology.

## 2 Materials and methods

### 2.1 Study population

In this study, we defined CHD/LD as abnormal configurations of thoracoabdominal organs, including the heart, lungs, liver, and spleen, in conjunction with any CHD classified by cardiac position, venous vessels, atria and ventricles, and ventricular outflow and great vessels. Children with SIT were also included. Patients with chromosomal abnormalities and CNVs were previously excluded through Chromosomal Microarray Analysis. Subsequently, we endeavored to recruit a total of 52 family trios, consisting both CHD/LD probands and their healthy parents. These individuals had sought medical care at the Children’s Hospital of Fudan University between January 2008 and August 2019. Trained personnel meticulously scrutinized the children’s medical records, encompassing outpatient hospital records, surgical records, and imaging reports (some of them can be found in Supplementary Figures S1–S7), and other relevant documents. This research received approval from the Ethics Committee at the Children’s Hospital of Fudan University, and informed consent was obtained from all participating guardians.

### 2.2 Identifying potential pathogenetic genes for CHD/LD

**FIGURE 1:**
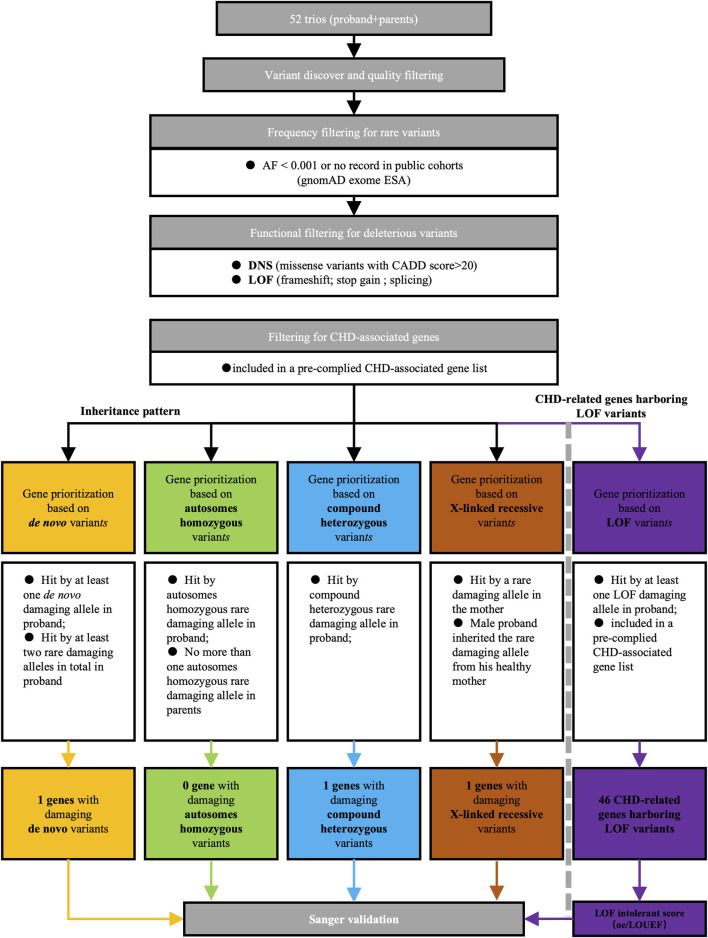
Screening strategy of candidate genes for CHD/LD pathogenicity. We first filtered out rare deleterious mutations based on the frequencies in the gnom_exome database and the CADD scores, of which there are two categories, DNS and LOF. Later, we screened candidate genes according to the inherited pattern in *de novo*, autosomes homozygous, compound heterozygous, and X-linked recessive, and then took those overlapping in LOF with previously reported CHD-related genes as LOF candidate genes, and finally verified them by sanger sequencing.AF, allele frequency; DNS, damaging nonsynonymous; LOF, loss of function; CADD, combined annotation dependent depletion; CHD, congenital heart disease.

To identify potential candidate genes implicated in CHD/LD, we conducted whole exome sequencing (WES) on 52 probands and their healthy parents, resulting in 21,556 high-quality variants after quality control. Our candidate gene filtering strategy (additional details are available in the Supplementary Material), as outlined in Figure 1, involved assessing variant rarity, deleteriousness, inheritance patterns, and gene function. The subsequent screening process for candidate genes adhered to predefined criteria. We initially filtered rare variants, defined as those with a variant frequency less than 0.001 or no record in the Genome Aggregation Database East Asian Genetic Ancestry Group (gnomAD_ESA), resulting in 11,312 rare variants. We then filtered rare deleterious variants, defined as variants among the rare missense variants with a Combined Annotation Dependent Depletion (CADD) score greater than 20 and all rare loss-of-function (LOF) variants (splicing, frameshift, and stopgain), yielding a total of 2,686 rare deleterious variants (1846 missense variants and 840 LOF variants). Among these, mutations in previously reported CHD genes (see Supplementary Table S1) (Li A. H. et al., 2019; Jin et al., 2017; Ellesøe et al., 2018; Zaidi and Brueckner, 2017) accounted for 48 LOF variants and 634 missense variants. Next, we applied a filtering process based on inheritance patterns, revealing one *de novo* mutations (in *DNAH2*), two compound heterozygous variants (in *DNAH2*), and one X-linked recessive variants (in *FLNA*). Furthermore, we analysis 46 CHD-related genes harboring 48 LOF variants (see Figure 6C). Subsequent functional and bioinformatics analyses were conducted on the 46 CHD-related genes harboring (see Figure 6D). Further filtering based on LOF intolerance scores.

#### 2.2.1 DNA extraction

We obstained peripheral blood DNA from the study subjects using Trizol reagent (Thermo Scientific, Waltham, MA, United States). The DNA was spectrophotometrically assessed using the NanoDrop 2000 (Thermo Scientific, Waltham, MA, United States) and its quality was observed through agarose gel electrophoresis. Subsequently, the samples were temporarily stored in a refrigerator at 4°C or −20°C for a short period, with the intention of conducting sequencing as promptly as possible.

#### 2.2.2 Whole exome sequencing

Exome libraries were generated using the NimbleGen’s SeqCap EZ Human Exome Library v2.0 Kit, a commercially available kit from Roche (Pleasanton, CA, United States). Subsequently, the prepared libraries underwent sequencing on the Illumina HiSeq X Ten platform, provided by Illumina Inc. (San Diego, CA, United States).

#### 2.2.3 Sequencing data preprocessing

To upload academic integrity, the following statement presents a rephrased version of the original text: The initial processing of raw sequencing data involved Adapter trimming and quality filtering, accomplished through the utilization of Cutadapt (v1.10) and FASTQC (v0.11.5). Subsequently, the processed data underwent alignment to the human reference genome (UCSC hg19) using bwa (v0.7.15) with default settings. Duplicate reads were identified through the MarkDuplicates tool in the Picard toolkit v2.5 (available at https://github.com/broadinstitute/picard) and were subsequently excluded from further analysis. The remaining reads underwent base quality recalibration using the BaseRecalibrator tool in the Genome Analysis Toolkit (GATK v3.8).

#### 2.2.4 Variant discovery and quality filtering

We adhered to the recommended best-practice pipeline for germline SNPs and indels outlined by GATK to carry out variant discovery and quality filtering. The detailed procedure is described in the following link: https://software.broadinstitute.org/gatk/best-practices/workflow?id=11145. In summary, the analysis comprised variant calling for each sample using HaplotypeCaller in GVCF mode, followed by joint genotyping of GVCF files for all samples. Subsequently, variant quality was evaluated and filtered using variant quality score recalibration (VQSR). To enhance specificity, variants with a minimal depth of less than 8x across all samples were excluded.

#### 2.2.5 Population frequency and functional annotation of variants

ANNOVAR was employed to annotate the variations, providing information on their functional and demographic frequency properties. The RefSeq gene model as the basis for this study. To filter for rare variants, allele frequencies from the Eastern Asian (ESA) population, as documented in the Genome Aggregation Database (gnomAD), were utilized. Variants with an allele frequency below 0.001 or those not documented in these public databases were classified as rare.

#### 2.2.6 Rare deleterious variants

Rare and deleterious variants were categorized into three groups: (1) all rare non-synonymous variants (with an allele frequency less than 0.001 in gnomAD_ESA and a CADD score greater than 20); (2) all rare loss-of-function variants (with an allele frequency less than 0.001 in gnomAD_ESA and causing frameshift, stop gain/loss, or splicing changes); and (3) a combination of rare non-synonymous and loss-of-function variants.

#### 2.2.7 ACMG classification

Following the evidence-based guidelines endorsed by the American College of Medical Genetics and Genomics (ACMG), all potentially damaging variants on the candidate genes were classified into five categories: pathogenic (P), likely pathogenic (LP), variant of uncertain significance (VUS), likely benign (LB), and benign (B).

#### 2.2.8 Functional enrichment analysis

The functional enrichment analysis and network visualization were conducted using GlueGO in Cytoscape software with default settings. The adjusted *P*-value threshold was set at 0.05, and the analysis utilized the Gene Ontology biological process (version: 25.05.2022).

#### 2.2.9 LOF intolerant score

For all CHD-related genes harboring LOF variants, the observed/expected (oe) and LOEUF scores were obtained from the gnomAD database. Candidate genes were then prioritized based on the database’s recommended criteria. Specifically, for the interpretation of Mendelian disease cases, it is suggested to use a LOEUF score < 0.6 as a threshold when necessary. Nonetheless, it is advisable to consider oe and LOEUF scores as continuous metrics rather than relying solely on a fixed cutoff.

#### 2.2.10 Sanger sequencing

All rare deleterious variants in candidate genes underwent validation through Sanger sequencing. To amplify the genomic region containing the variant and its surrounding ±10 bp, we designed primers using Primer3Web v4.1.0 (http://primer3.ut.ee/). The polymerase chain reaction (PCR) was employed for amplification, followed by agarose gel electrophoresis and sequencing using an ABI 3730XL Genetic analyzer (Applied Biosystems, Foster City, CA, United States).

### 2.3 Statistical analysis

Statistical calculations were performed by SPSS 23.0. Count data were expressed as the mean (standard deviation) or median (interquartile range). The criterion for significance was set at a p-value less than 0.05. Categorical variables were compared using the chi-square test, continuous variables were assessed using the Kruskal-Wallis test, and for count variables with theoretical values of 10, Fisher’s test was employed.

## 3 Results

### 3.1 Demographic and clinical characteristics of CHD/LD

This study included 52 probands diagnosed with CHD/LD (Table 1), with a male-to-female ratio of 1.48:1 (31:21), suggesting a potential male susceptibility to this condition. The average age of diagnosis was 2.58 months. The most prevalent anatomical abnormalities were as follows: dextrocardia in 57.59% (30/52) of cases for cardiac position, bilateral superior vena cava in 44.23% (23/52) for venous anomalies, atrial septal defects in 61.54% (32/52) within atrial and ventricular structures, right aortic arch in 57.69% (30/52) for ventricular outflow and great vessels, left-side liver in 38.46% (20/52) of cases, right-sided spleen in 46.15% (24/52), and left-right reversal of pulmonary structures in 25.00% (13/52) of individuals.

**TABLE 1:** Demographic and clinical characteristics of CHD/LD probands.

	Number	Percentage (%)
Gender		
Male	31	59.62%
Female	21	40.38%
Age in diagnosed (months)	2.58 (0.33–7.63)	
Cardiac position		
Levocardia	20	38.46%
Dextrocardia	30	57.59%
Mesocardia	2	3.85%
Venous anomalies		
Interrupted inferior vena cava	1	1.92%
Bilateral superior vena cava	23	44.23%
Anomalous pulmonary venous return	7	13.46%
Atria and ventricles		
Atrioventricular septal defect	15	28.84%
Single ventricle morphology	22	42.31%
Atrial septal defect	32	61.54%
Ventricular septal defect	26	50.00%
Atrioventricular valve stenosis/atresia	8	15.38%
Ventricular outflow and great vessels		
Double outlet right ventricle	18	34.62%
Transposition of the great artery	20	38.46%
Pulmonary stenosis/atresia	18	34.62%
Truncus arteriosus	0	0
Aortic stenosis	0	0
Coarctation of the aorta	1	1.92%
Right aortic arch	30	57.69%
Liver		
Left-sided	20	38.46%
Middle	17	32.69%
Spleen		
Right-sided	24	46.15%
Asplenia	16	30.77%
Polysplenia	5	9.62%
Lung		
Invert	13	25.00%
Left isomerism	5	9.62%
Right isomerism	6	11.54%

### 3.2 Identifying potential pathogenetic genes for CHD/LD

#### 3.2.1 Candidate genes in *de novo* variants

**FIGURE 2:**
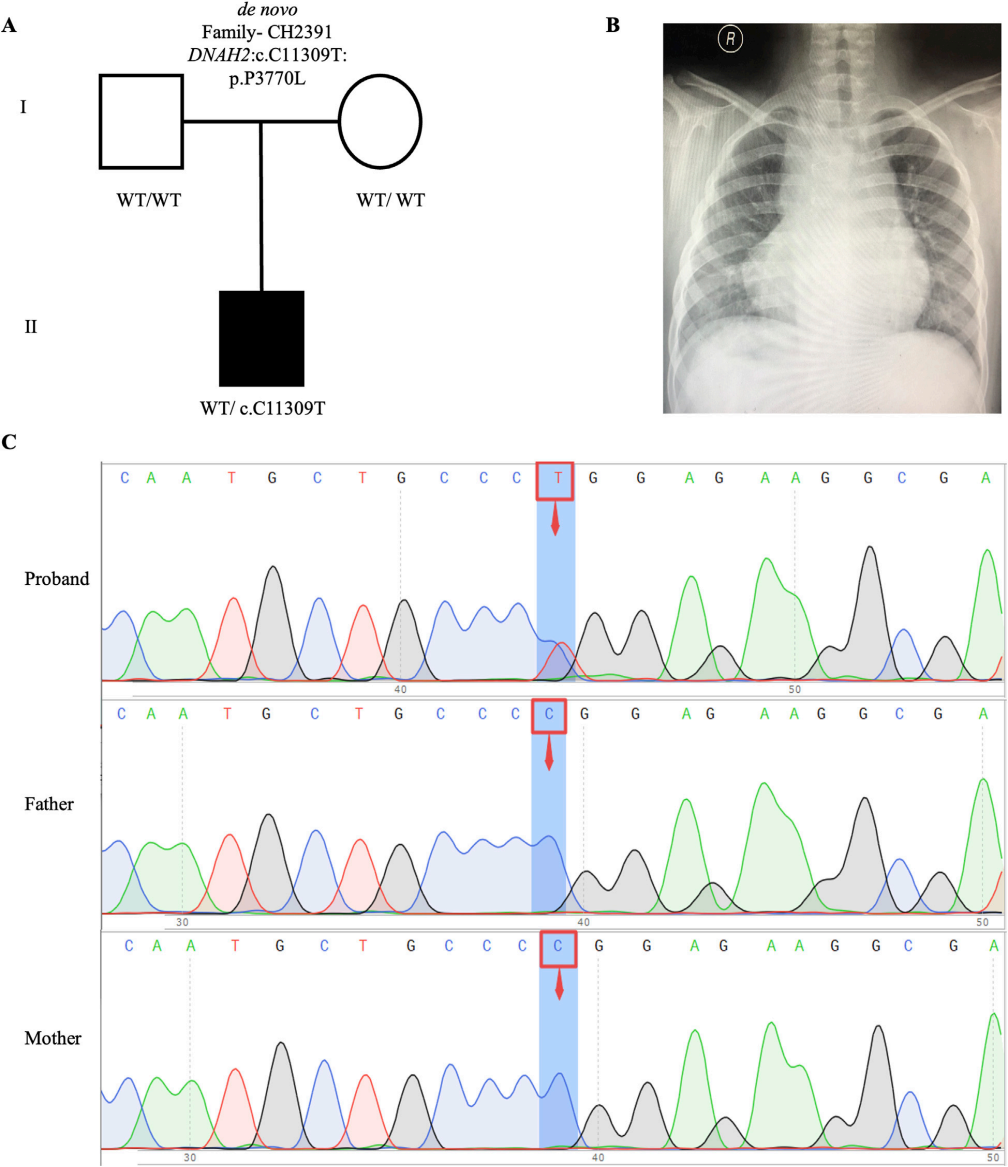
*De novo* variant in CH2391 family trio **(A)** The pedigree chart of the CH2391 family, in which the proband carry the *de novo* variant (*DNAH2*: c.C11309T). **(B)** Chest radiograph showing dextrocardia in the proband CH2391. **(C)** Sanger sequencing results show that the *de novo* variant (*DNAH2*: c.C11309T) was found in the proband, while his parents did not carry this variant. The blue background indicates the nucleotide change from C to T in the proband at position 11,309.

We identified one potential candidate genes *DNAH2*, harboring a *de novo* variant (Table 2). *DNAH2*, a cilia gene previously associated with sperm flagellum function and spermatogenic failure (Li Y. et al., 2019), exhibited *de novo* variant (CH2391: NM_020877: c. C11309T: p. P3770L) and compound heterozygous variants (CH5033: NM_020877: c. C8527T: p. P2843S/c. C10808T: p. A3603V). Notably, these *DNAH2* variants were located on highly conserved amino acids within critical structures of the heavy chain of ciliary dynein, including AAA4, AAA5, and AAA6 (Figures 6A, B), which play a pivotal role in generating the force propelling the movement of ciliary dynein through conformational changes (Roberts et al., 2013; King, 2021). Intriguingly, *DNAH2* exhibited the highest variant frequencies among all candidate genes, underscoring its potential candidacy. Furthermore, two probands carrying the *DNAH2* variant consistently presented with dextrocardia in their X-ray chest records (Figures 2B, 4B), providing support for the hypothesis that abnormal *DNAH2* gene function may play a role in CHD/LD development. The pedigree of family CH2391 and Sanger sequencing can be found in Figures 2A, C.

**TABLE 2:** Candidate genes prioritized based on inheritance mode.

Gene	Position	Putatively damaging variant	Functional category	Allele frequency in GnomAD_EAS	CADD	Inheritance mode	ACMG classification	Envidence	Proband:GT	Father:GT	Mother:GT
DNAH2	chr17:7726926	NM_020877:c.C11309T:p.P3770L	missense	5.01E-05	26.5	*de novo*	VUS	PM1	CH2391:0/1	CH2391F:0/0	CH2391M:0/0
FLNA	chrX:153578474	NM_001456:c.G7234A:p.V2412I	missense	—	28.5	X-linked recessive	VUS	PM1; PM2; PP3	CH0175:1/−	CH0175F:0/−	CH0175M:0/1
DNAH2	chr17:7702004	NM_020877: c.C8527T:p.P2843S	missense	7.07E-04	22	Compound	VUS	PM1	CH5033:0/1	CH5033F:0/0	CH5033M:0/1
	chr17:7722374	NM_020877:c.C10808T:p.A3603V	missense	2.04E-04	23.2	heterozygous	VUS	PM1	CH5033:0/1	CH5033F:0/1	CH5033M:0/0

Position, Chromosome and Position of the variant; GnomAD_EAS, genome aggregation database east asian genetic ancestry group; CADD, combined annotation dependent depletion score; Proband:GT, Proband ID:Genotype (0/1:heterozygous; 1/1:homozygous); ACMG classification, The classification of the variant based on ACMG guidelines; P, pathogenic; LP, likely pathogenic; VUS, variant uncertain significance; LP, likely benign; B, benign; Envidence, Envidence for ACMG classification.

#### 3.2.2 Candidate genes in X-linked recessive variants

**FIGURE 3:**
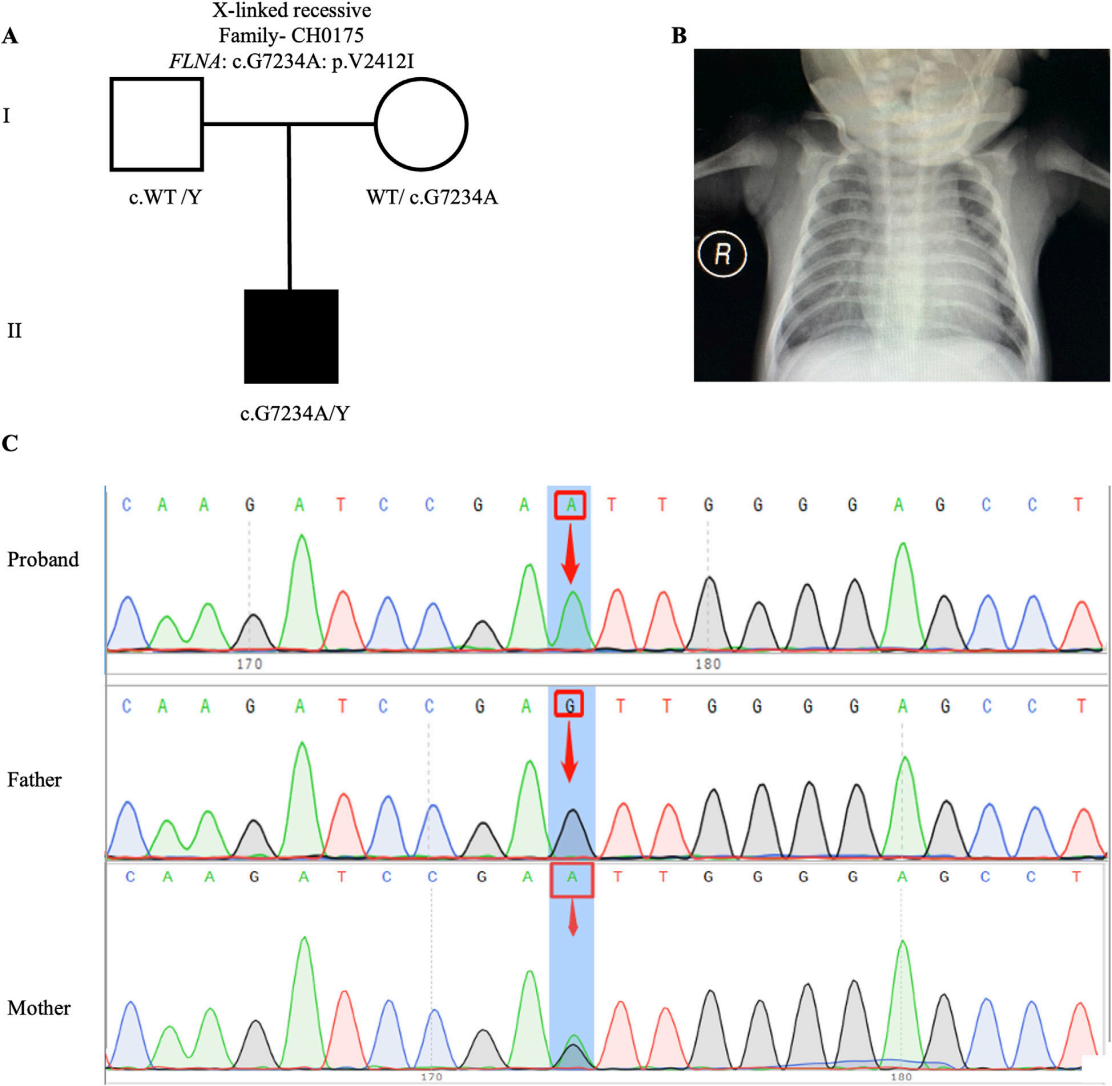
X-linked recessive variants in CH0175 family trio **(A)** The pedigree chart of the CH0175 family, in which the proband carry the X-linked recessive variant (*FLNA*: c. G7234A). **(B)** Chest radiograph showing levocardia in the proband CH0175. **(C)** Sanger sequencing results show that the X-linked recessive variants (*FLNA*: c. G7234A) was found in the proband, while his mother carry heterozygous variant. The blue background indicates the nucleotide change from G to A in the proband at position 7,234.

Previous studies have proposed an X-linked recessive inheritance pattern for CHD/LD (Antony et al., 2022), a hypothesis further supported by the observed male-to-female ratio of 1.48:1 in our cohort. We identified one candidate gene carrying X-linked recessive variant: *FLNA* (CH0175: NM_001456: c. G7234A: p.V2412I). *FLNA* has been previously reported as an X-linked recessive genetic factor associated with FG syndrome and CHD (Unger et al., 2007). This study, along with a 2020 Chinese population-based CHD/LD study, indicates that *FLNA* is the causative gene for this disease (Liang et al., 2020). The pedigree, chest X-ray, and Sanger sequencing results of the proband CH0175 can be found in Figures 3A–C.

#### 3.2.3 Candidate genes in compound heterozygous variants

**FIGURE 4:**
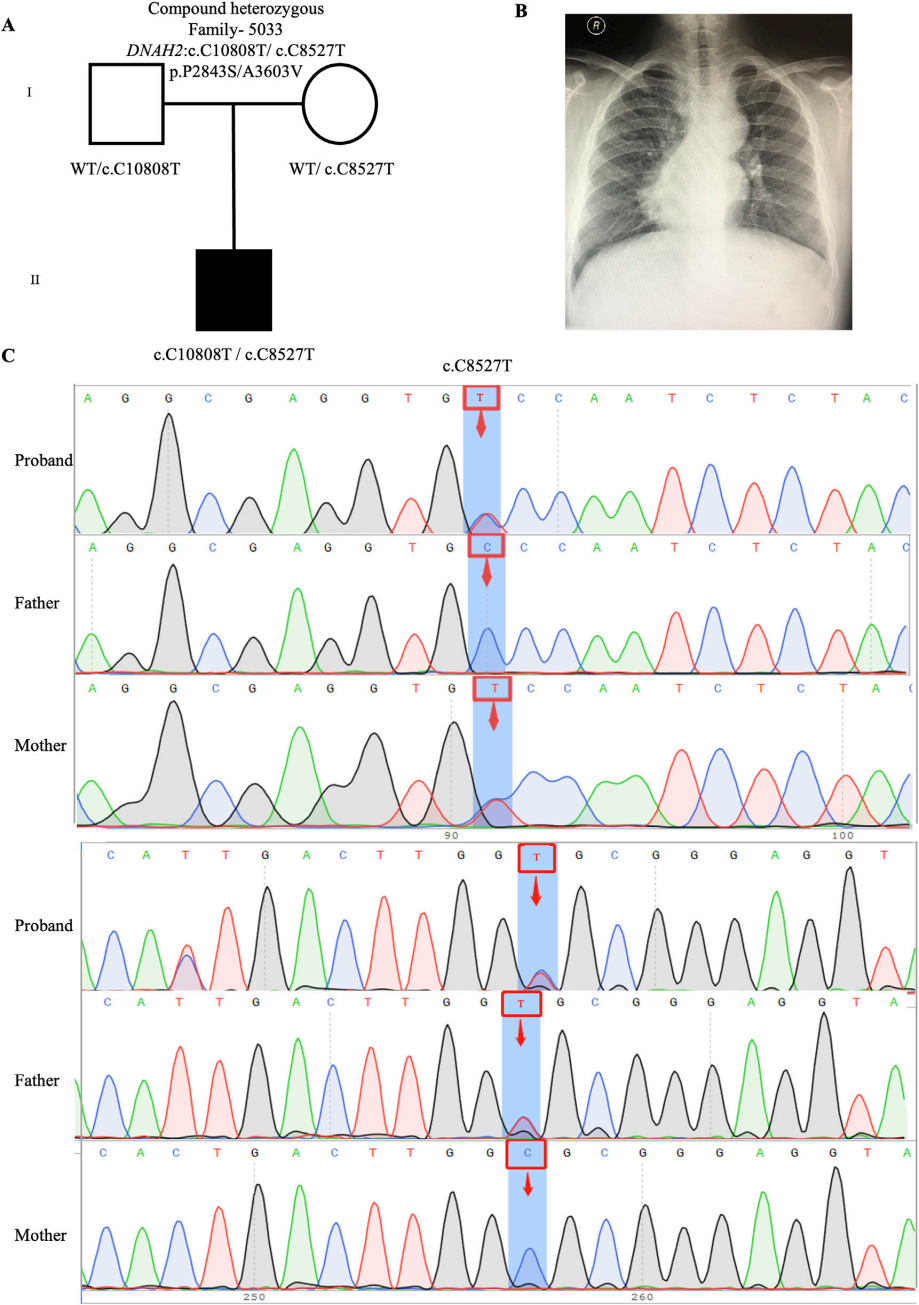
Compound heterozygous variants in CH5033 family trio **(A)** The pedigree chart of the CH5033 family, in which the proband carry the compound heterozygous variants (*DNAH2*: c.C10808T/c.C8527T). **(B)** Chest radiograph showing levocardia in the proband CH5033. **(C)** Sanger sequencing results show that the compound heterozygous variants (*DNAH2*: c.C10808T/c.C8527T) was found in the proband, while each his parents carry a heterozygous variant. The blue background indicates the nucleotide change from C to T in the proband at position 8,527, and from C to T in the proband at position 10,808.

One candidate genes harboring compound heterozygous variants were identified: *DNAH2* (CH5033: NM_020877: c.C8527T: p.P2843S/c.C10808T: p.A3603V), Remarkably, among all candidate genes, *DNAH2* exhibited one of the two highest frequencies of variants. Previous reports have linked compound heterozygosity in *DNAH* family members, such as *DNAH9* (Fassad et al., 2018), *DNAH5* (Nothe-Menchen et al., 2019), and *DNAH11* (Xia et al., 2021), to the development of CHD/LD. As mentioned in the section on *de novo* candidate genes, both probands in our study carrying rare deleterious *DNAH2* variants exhibited dextrocardia (Figures 2B, 4B). This study represents the first to associate *DNAH2* with CHD/LD. The pedigree and Sanger sequencing results of the proband CH5033 can be found in Figures 4A–C.

#### 3.2.4 CHD-related genes harboring LOF variants

**FIGURE 5:**
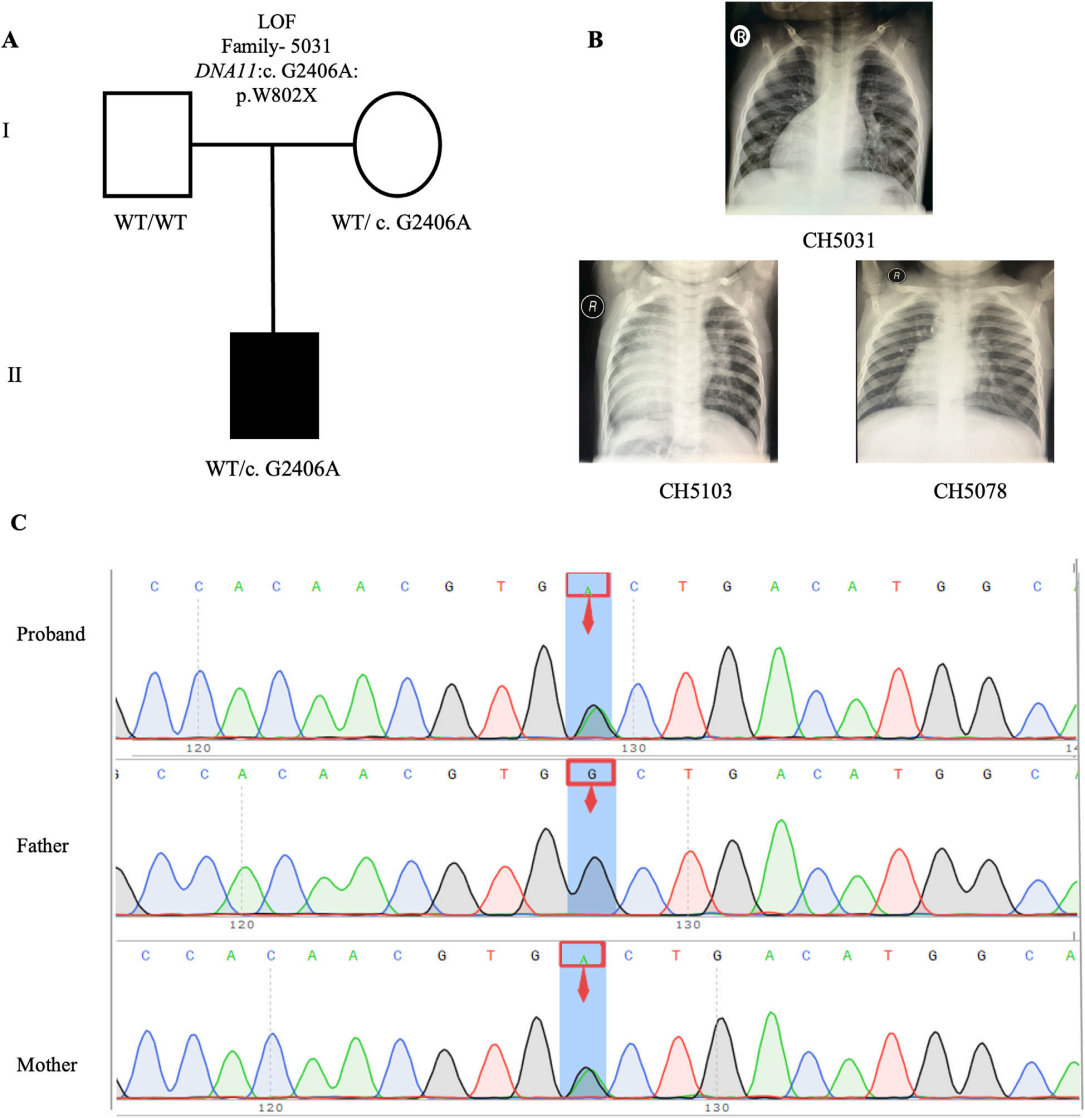
LOF variant in CH5031 family trio **(A)** The pedigree chart of the CH5031 family, in which the proband carry the LOF variants (*DNAH11*: c. G2406A). **(B)** Chest radiograph showing dextrocardia in the proband CH5031, CH5103, and CH5078. **(C)** Sanger sequencing results show that the LOF variant (*DNAH11*: c. G2406A) was found in the CH5031, while his mother carries same variant. The blue background indicates the nucleotide change from G to A in the CH5031 at position 2,406.

**FIGURE 6:**
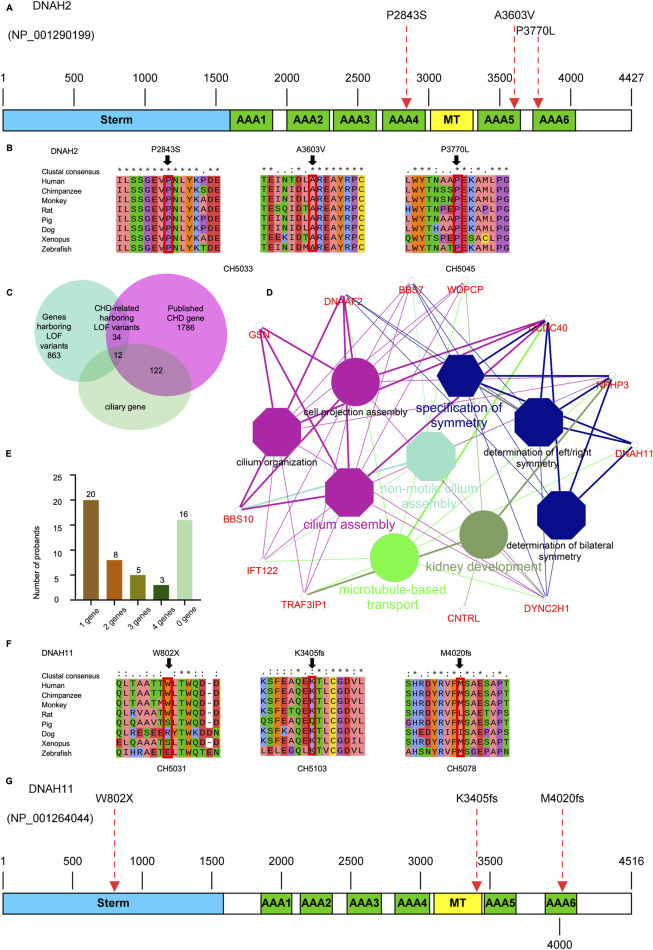
Variants in cilia-related genes are associated with the development of CHD/LD **(A, G)**, the schematic diagrams of the protein functional domains of *DNAH2* and *DNAH11*, respectively, and the dashed red arrows indicate the mutation sites. **(B, F)**, Sequence comparison of different species of *DNAH2* and *DNAH11* showing the high conservation of the mutant amino acids; **(C)**, the overlap between LOF genes harboring rare LOF variants that have been reported as both CHD-associated and cilia-associated; **(D)**, enrichment analysis revealed that CHD-related genes harboring LOF variants were significantly overrepresented in cilia-associated pathways. The significance threshold for enrichment was set at a Bonferroni-adjusted multiple test *P* value of less than 0.05; **(E)**, number of candidate genes affected in the probands, and the number of probands in the upper part of the bar.

Upon intersecting all 840 rare deleterious LOF variants with a collection of 1786 published CHD-related genes (Supplemental Method and Supplementary Table S1), we identified 46 CHD-related genes harboring LOF variants (Supplementary Table S2). Subsequently, we scrutinized all the rare deleterious variants in these 46 CHD-related genes harboring LOF variants. Notably, we observed a higher proportion (12 out of 46, 26.1%) of these genes linked with cilia-related functions, compared to only 7.5% (134) of the 1786 reported CHD genes (Figure 6C). This finding emphasizes the crucial role of cilia genes in the pathogenesis of CHD/LD. Through gene enrichment analysis, we detected 12 cilia-related genes (*BSS10*, *BSS7*, *CCDC40*, *CNTRL*, *DNAAF2*, *DNAH11*, *DYNC2H1*, *GSN*, *IFT122*, *NPHP3*, *TRAF3IP3*, and *WDPCP*) enriched in various cilia-related pathways, including cilium assembly, determination of left-right symmetry, intraciliary transport, and microtubule transport (Figure 6D). These results align with previous studies emphasizing the pivotal role of cilia in the development of left-right structures in organisms (Zimmerman and Yoder, 2015; Zhou and Roy, 2015). Notably, variants in *DNAH11* exhibited one of the two highest frequencies. We identified three variants in three probands (CH5031: NM_001277115: c. G2406A: p.W802X; CH5103: c.12058_12059del: p.M4020fs*; CH5078: c.10214_10217del: p. K3405fs*) (Supplementary Table S2). Remarkably, three probands carrying *DNAH11* variants exhibited dextrocardia (Figure 5B). The pedigree and Sanger sequencing results of the proband CH5033 can be found in Figures 5A, C. The conservation of the *DNAH11* LOF variants and the schematic diagram of the domains containing the three mutation sites can be found in Figures 6F, G.

#### 3.2.5 Post-hoc pathogenicity analysis of candidate variants

We performed a *post hoc* pathogenicity analysis on all variants identified through genetic pattern screening, including one *de novo* variants (*DNAH2*), two compound heterozygous variants (*DNAH2*), one X-linked recessive variants (*FLNA*), and three variants of CHD-related cilia gene *DNAH11*. The healthy control cohort was selected from the gnom_AD East Asian population database and Han Chinese Genomes database, and the analysis results are presented in Table 3. The results indicate that, except for the *DNAH2* variant (c.C8527T: p.P2843S, *p*-values = 0.0809 and 0.0834), which shows no significant difference in both databases, all candidate pathogenic variants demonstrate significantly higher mutation frequencies compared to the healthy population. Given that the variant are inherited by the proband through a compound heterozygous pattern alongside the *DNAH2* variant (c.C10808T: p.A3603V, *p*-values = 0.0315 and 0.0253), its pathogenic potential remains substantial. Moreover, all three variants of the CHD-related genes *DNAH11* (c.G2406A: p.W802X; c.12058_12059del: p.M4020fs*; c.10214_10217del: p. K3405fs*, *p* = 0.019) are inherited from the healthy parents of the proband, yet these variants clearly disrupt protein structure, warranting further experimental validation of their pathogenicity.

**TABLE 3:** *Post-hoc* pathogenicity analysis of candidate variants in probands compared to the healthy population.

Gene	Position	Putatively damaging variant	Inheritance mode	Alle count/alle number in our cohort probands^a^	Alle count/alle number in gnom_AD East Asia populations^b^	P-value	Alle count/alle number in Han Chinese genomes^b^	P-value
*DNAH2*	chr17:7726926	NM_020877.5:c.C11309T:p.P3770L	*De novo*	1/104	1 + 0/(19,954 + 208)	**0.0103**	**—**	**0.0166**
*FLNA*	chrX:153578474	NM_001456.4:c.G7234A:p.V2412I	X-linked recessive	1/52	—	**0.0048**	**—**	**0.0085**
*DNAH2*	chr17:7702004	NM_020877.5: c.C8527T:p.P2843S	Compound heterozygous	1/104	13 + 1/(18,392 + 208)	0.0809	33 + 1/(41,916 + 208)	0.0834
*DNAH2*	chr17:7722374	NM_020877.5:c.C10808T:p.A3603V	Compound heterozygous	1/104	4 + 1/(19,634 + 208)	**0.0312**	5 + 1/(28,398 + 208)	**0.0253**
*DNAH11*	chr7:21630934	NM_001277115.2:c.G2406A:p.W802X	—	1/104	—	**0.0186**	**—**	**0.0166**
*DNAH11*	chr7:21912981	NM_001277115.2:c.12058_12059del:p.M4020fs*	—	1/104	—	**0.0186**	**—**	**0.0166**
*DNAH11*	chr7:21847548	NM_001277115.2: c.10215_10218del:p.K3405fs*	—	1/104	—	**0.0186**	**—**	**0.0166**

^a^ The variants on autosomes have two alleles, so the alle count of proband is 52 * 2 = 104. Variants on the X chromosome in males have only one allele, so the alle count of proband is 52 * 1 = 52.

^b^ The number following the plus sign in the numerator and denominator represents the allele count of healthy parents in this study. The alle count of parents is 52 * 2 + 52 * 2 = 208 or 52 * 2 + 52 * 1 = 156. Since some variant locus is not present in the gnom_AD, database, the denominator is taken as the minimum allele number in the table, which is 10,904 + 208 or 10,904 + 156; Similarly, for variant loci not present in the Han Chinese Genomes database, the denominator is taken as the minimum allele number in the table, which is 5,998 + 208.

## 4 Discussion

We analyzed 52 unrelated Chinese family trios, each consisting of probands with CHD/LD and their healthy parents. WES was performed on these 52 core families, followed by a filtering strategy. We identified two *de novo* variants (*DNAH2*), two compound heterozygous variants (*DNAH2*), 1 X-linked recessive variants (*FLNA*). Notably, the ciliary genes *DNAH2* was the most significant genes harboring pathogenic variants, occurring three times in the variant screening. Additionally, enrichment analysis of the 46 CHD-related genes harboring LOF genes revealed that 12 genes were significantly associated with ciliary function. These results underscore the pivotal role of ciliary genes in the pathogenesis of combined LD and CHD. To our knowledge, this study represents the largest investigation of CHD/LD family trios in the Chinese population, identifying 11 candidate genes and 46 CHD-related genes harboring LOF variants and expanding the potential spectrum of causative genes. The interpretation of these genes is challenging due to their intricate genetic background (Li A. H. et al., 2019), with only a subset of cases being explained by single-gene pathogenicity. In our study of 52 probands (Figure 6E), 20/52 (38.46%) were found to be associated with a single related gene, while 16/52 (30.77%) exhibited links to two or more these genes, and 16/52 (30.77%) probands did not have any implicated candidate genes. These findings reveal the complex genetic landscape of CHD/LD, which comprises oligogenic or multi-gene interactions that contribute to the disease.

Numerous genes have been associated with CHD/LD in prior research. For example, Hornef et al. reported that *DNAH5* is a significant genetic factor for PCD and heterotaxy in 109 PCD families from North America and Europe (Hornef et al., 2006). Similarly, Ware et al. analyzed 20 familial and 145 sporadic heterotaxy cases across diverse ethnicities, revealing that *ZIC3* is instrumental in the disease’s pathogenesis (Ware et al., 2004). Mohapatra et al. identified a strong correlation between *NODAL* variants and this disease in their investigation of 206 patients (Mohapatra et al., 2008). Fassad et al. reported that variants in *DNAH9* are pathogenic factors for this disease (Fassad et al., 2018). Qin et al. also linked *de novo* variants in *MMP21* with the disease’s incidence (Qin et al., 2022). Other implicated genes include *CCDC40*, *GDF1*, and *ACVR2B*, among others (Gao et al., 2019; Kosaki et al., 1999; Sugrue and Zohn, 2017). In this study, we also scrutinized the variants of these reported genes harboring pathogenic variants associated with CHD/LD. Specifically, we discovered (data not shown) that 12 patients carried *DNAH5* variants, 7 patients carried *CCDC40* variants (including one LOF variant), 13 patients carried *DNAH9* variants, 2 patients carried *NODAL* and *GDF1* variants, respectively, 1 patient carried an *ACVR2B* variant, and no patients carried *ZIC3* and *MMP21* variants. However, these reported pathogenetic genes did not appear in the results of this study. There are two primary reasons for this. Firstly, our study population is composed of the Chinese population, whereas previous studies have focused on other populations. Secondly, the variants of these candidate genes did not meet the screening criteria of this study (see Figure 1), such as the inheritance patterns and definition of rare deleterious variants.

In this study, two candidate genes identified based on inheritance patterns *de novo* (*DNAH2*), X-linked recessive (*FLNA*), and compound heterozygous (*DNAH2*), are reported for the first time, thereby expanding the spectrum of candidate genes for CHD/LD. And, all CHD-related genes harboring LOF variants identified in this study are listed in Supplementary Table S2. Among them, a variant in *DNAH11* (c.G2406A: p.W802X) has been previously associated with this disease. While variants in *CCDC40*, *DNAAF2*, *DYNC2H1*, *GDF1*, *IFT122*, *NPHP3*, and *WDPCP* have also been reported in association with this condition, the specific variants identified in our cohort are reported here for the first time. Although the specific variants identified in our study are reported for the first time. Furthermore, the LOF variants identified in the low-LOUEF score genes *FAM91A1*, *INTS8*, and *PDE2A* have not been previously linked to congenital heart disease and are newly reported in this study.

Nevertheless, it is essential to clarify that our filtering of CHD-related genes harboring LOF variants and candidate LOF genes differ from the previously mentioned candidate genes, which were selected based on inheritance patterns. All filtered LOF variants were inherited from phenotypically normal parents, complicating traditional dominant/recessive classification and suggesting potential oligogenic inheritance or incomplete penetrance. Given the intricate genetic background of this disease and the profound impact of LOF variants on gene functions. As well as a pivotal research question in genetic counseling revolves around whether the offspring of first-degree relatives carrying the same LOF variant are susceptible to developing the disease. To address this, we conducted an alternative filtering method for LOF variants by intersecting them with previously reported CHD-related genes to identify potential pathogenic LOF genes. This approach yielded 46 CHD-related genes harboring LOF variants for subsequent functional analysis. Remarkably, the cilia gene *DNAH11* demonstrated one of the two highest variant frequencies, and 12 out of the 46 candidate genes (26.1%) are associated with ciliary function. Initial reports of Kartagener syndrome revealed the relationship between respiratory cilia and left-right structural anomalies (Kartagener, 1933), which has since expanded to a broader range of conditions known as primary ciliary dyskinesia (PCD) (Horani and Ferkol, 2021). Subsequent studies have established the crucial function of cilia in the development of embryonic left-right structures. The ciliated epithelial tissue of the left-right organizer performs a key role in transforming the body part from symmetrical to asymmetrical (Hirokawa et al., 2006), achieved through the coordinated activity of motile and sensory cilia on the LRO (Blum et al., 2007). Motile cilia create a left-to-right fluid flow through clockwise rotation, sensed by peripheral sensory cilia (Yoshiba et al., 2012; Nonaka et al., 1998; Takamatsu et al., 2013). Disruption of this process results in severe heterotaxy (Little and Norris, 2021). Our results reaffirm the significant involvement of cilia-related genes in CHD/LD pathogenesis (Dasgupta and Amack, 2016).

## 5 Limitation

The lack of laboratory validation for pathogenicity may compromise the reliability of our findings. Techniques using gene-targeted mutations in model organisms and stem cells could offer deeper insights into the molecular mechanisms underlying CHD/LD pathogenesis in future studies. Furthermore, although we analyzed the pathogenicity of candidate variants with the healthy population from the both gnom_AD database and Han Chinese Genomes database in our *post hoc* pathogenicity analysis (see Table 3), it remains necessary to utilize local healthy individuals and sporadic isolated CHD patients as controls to eliminate confounding factors. Finally, due to the limitations of whole-exome sequencing, certain genomic regions, such as mitochondrial genes, are not comprehensively covered. Additional research will require a larger sample size and the inclusion of both an isolated CHD control group and a healthy control group.

## 6 Conclusion

In summary, our investigation delved into the genetic profile of CHD/LD within Chinese populations, revealing previously undisclosed candidate genes and broadening the range of potential disease-associated genes. Notably, *DNAH2* exhibited the highest mutation frequency among all candidate genes. In addition, cilia-related genes collectively accounted for 26.1% of CHD-related genes harboring LOF variants. This collective finding underscores the pivotal role of cilia in the manifestation of this disease. The insights gained from this research contribute valuable knowledge regarding the genetic origins of CHD/LD in the Chinese population, carrying potential implications for genetic counseling and prenatal preventive measures.

## Data Availability

The data presented in the study are deposited in the Genome Sequence Archive repository, accession number HRA006883.
