# Supplementary material for: Identification of candidate genes harboring pathogenic variants in congenital heart disease and laterality defects in Chinese population
Source: Front Genet. 2025 May 8;16:1582718. doi: 10.3389/fgene.2025.1582718 (PMC12095028; doi:10.3389/fgene.2025.1582718)
Supplement: Supplementary file 2 [file DataSheet1.docx]

Supplementary Material

dentification of Potential Pathogenic Genes in Congenital Heart Disease and Laterality Defects in Chinese population

Jinxin Wang^1,2†^, Weicheng Chen^1,2†^, Xianghui Huang^1,2,4^, Han Gao^1,2^, Zhiyu Feng^1,2^, Chaozhong Tan^1,2^, Quannan Zhuang^1,2^, Yuan Gao^1,2^, Shaojie Min^1,2^, Yuquan Lu^1,2^, Feizhen Wu^1^, Maoxiang Qian^1,3^, Weili Yan^1,2,3^, Wei Sheng^1,2,3*^, Guoying Huang^1,2,3 *^

*** Correspondence:**Wei Sheng
sheng_wei@fudan.edu.cn

Guoying Huang
gyhuang@shmu.edu.cn

# Supplementary Data

## **Expanded Material and Method**

Screening strategy for candidate *de novo* genes

1. The parents do not carry this mutation

2. Among the rare deleterious genes, number of mutations in this gene≥2

Screening strategy for candidate autosomal homozygous genes

1. The gene are located on the autosomal chromosome

2. The parents do not carry the mutation or are heterozygous in this gene

3. The probands carry the autosomal homozygous mutations

Screening strategy for candidate compound heterozygous genes

1. Both parents carry different heterozygous mutations in this gene

2. The probands inherit one heterozygous mutation from each parent

Screening strategy for candidate X-linked recessive genes

1. Gene is located in a non-pseudo-autosomal region of the X chromosome

2. Mother carries a mutation in this gene

3. Male proband inherits this mutation from his mother

Screening strategy for CHD-related genes harboring LOF genes

1. Mutations are loss-of-function frameshift, stop gain/loss, and splicing

2. Included in a list of 1786 published CHD-causing genes (see Supplemental Table1) (1-4)

# Supplementary Figures and Tables

## Supplementary Figures


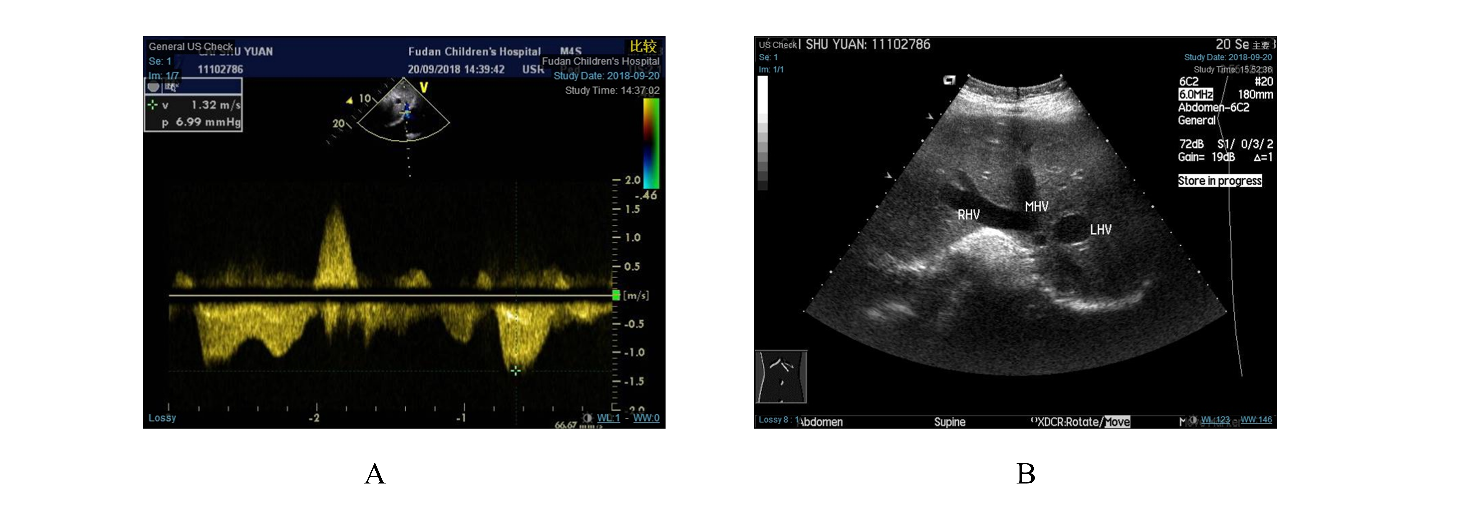


**Supplementary Figure 1.** Echocardiography following Glenn procedure and abdominal ultrasonography(left-liver) for CH2391 carrying *de novo* variant (*DNAH2*: c.C11309T, p.P3770L)


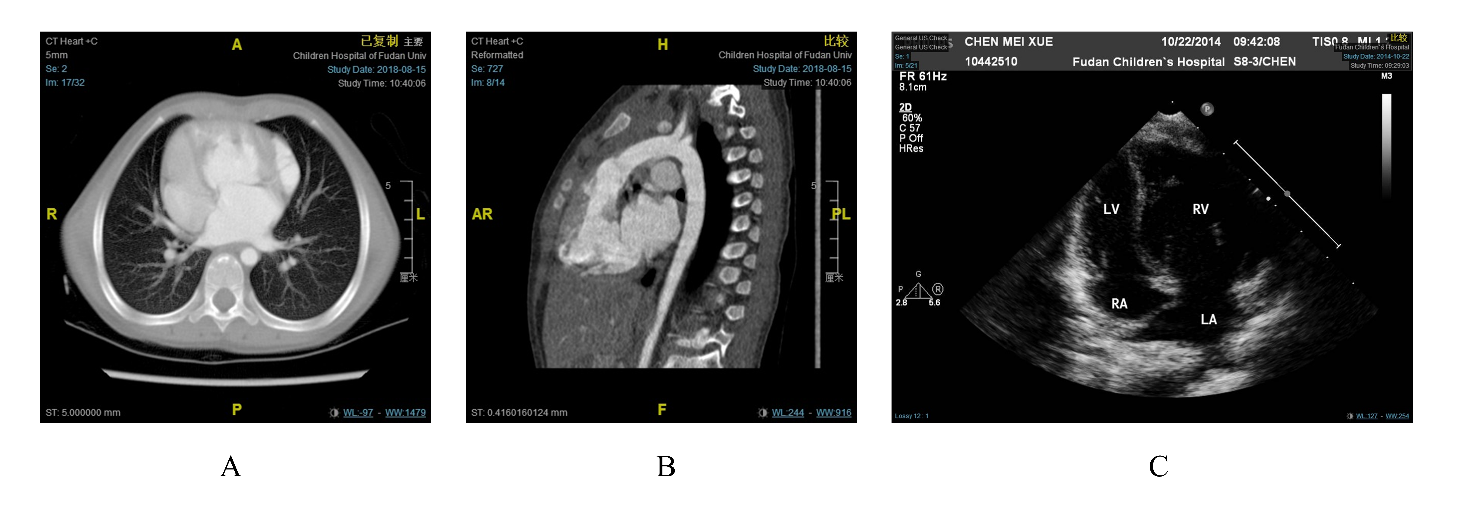


**Supplementary Figure 2.** Chest CT and Echocardiography Results for CH2319 carrying autosomal homozygous variants (*USP45*: c.C2222T/c.C2222T, p.S741L)


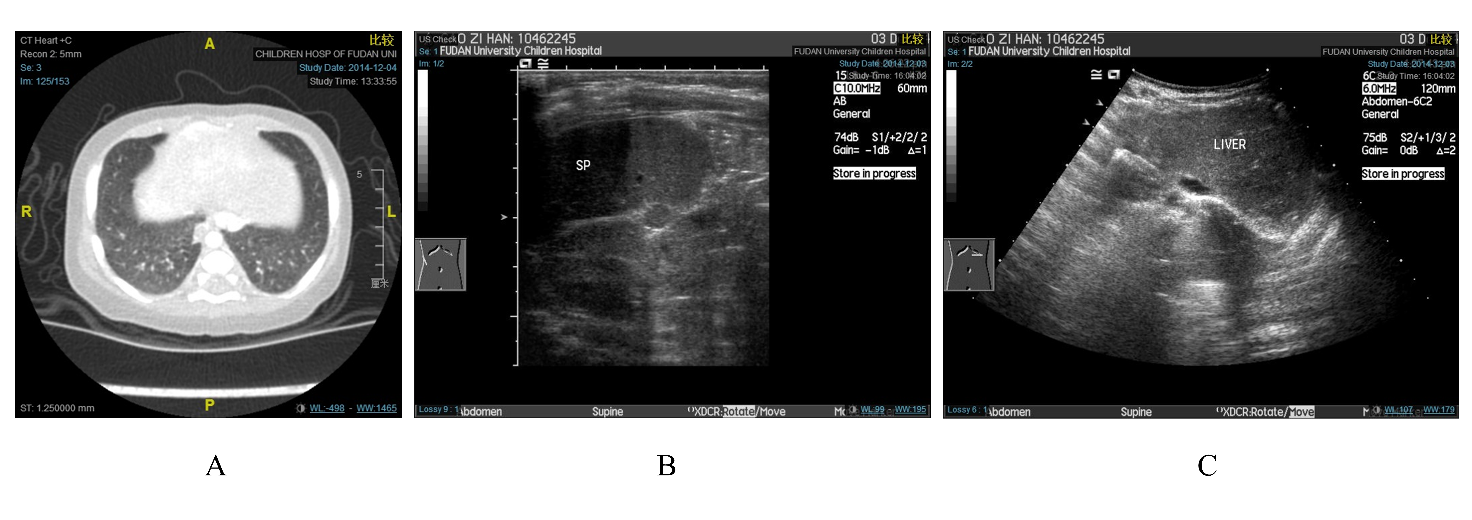


**Supplementary Figure 3.** Chest CT and Abdominal Ultrasonography Results for CH2134 carrying X-linked recessive variant (*RNF128*: c.G436A: p.G146S)


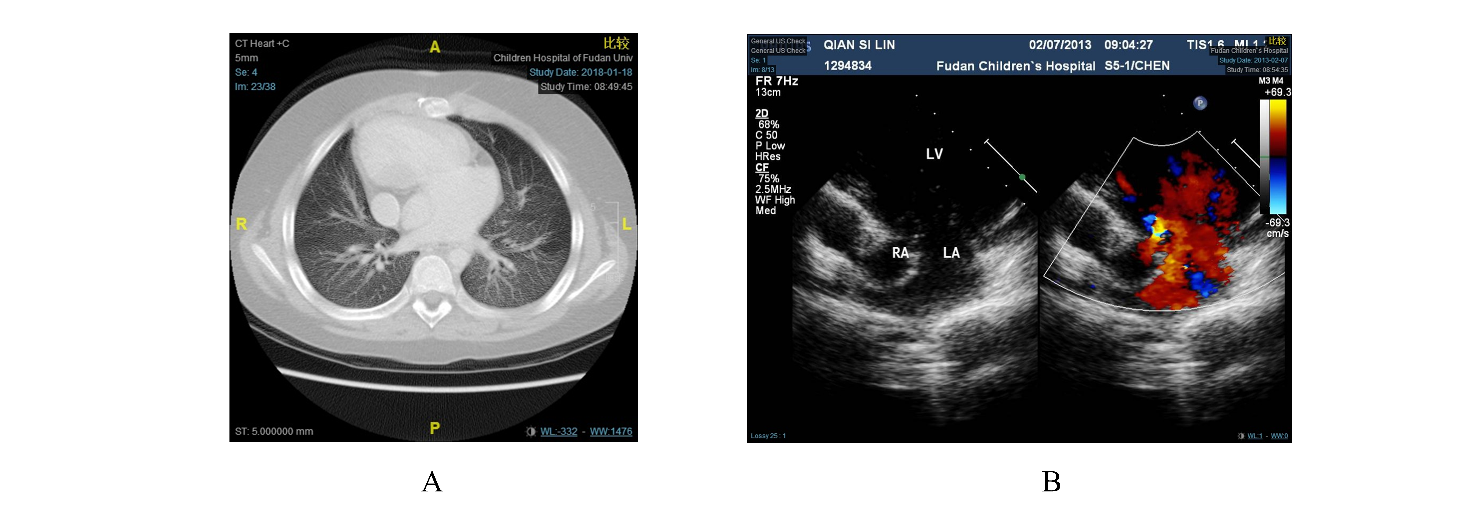


**Supplementary Figure 4.** Chest CT and Echocardiography Results for CH5033 carrying compound heterozygous variants (*DNAH2*:c.C10808T/ c.C8527T p. P2843S/A3603V)


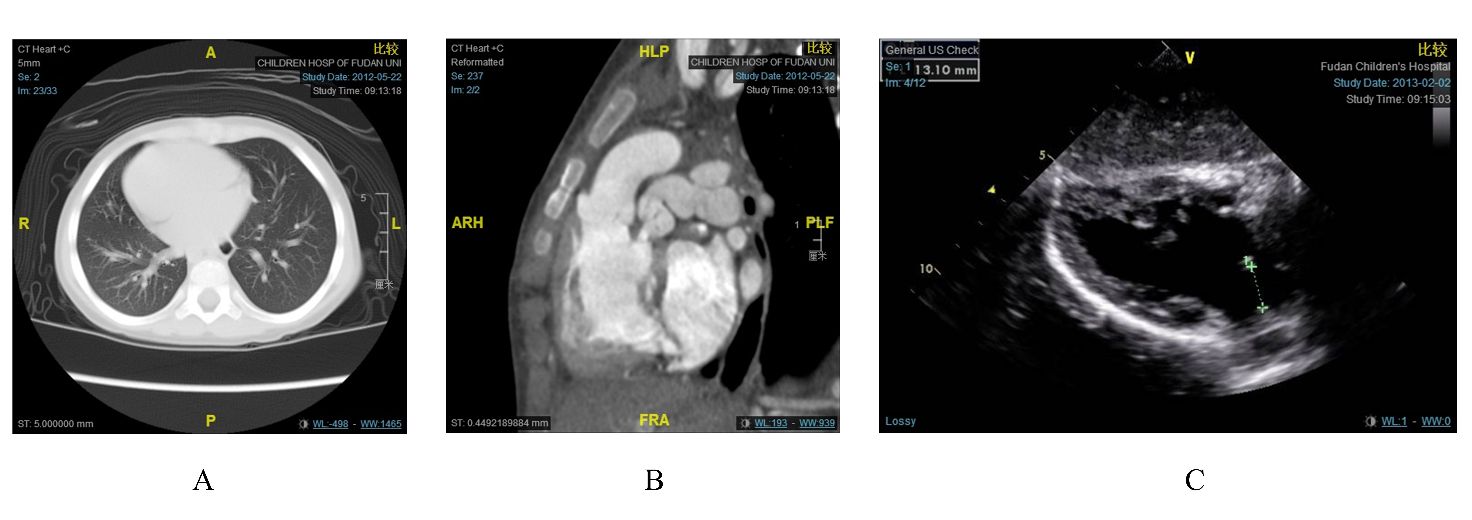


**Supplementary Figure 5.** Chest CT and Echocardiography Results for CH5031 carrying LOF variant (*DNA11*: c. G2406A: p.W802X)


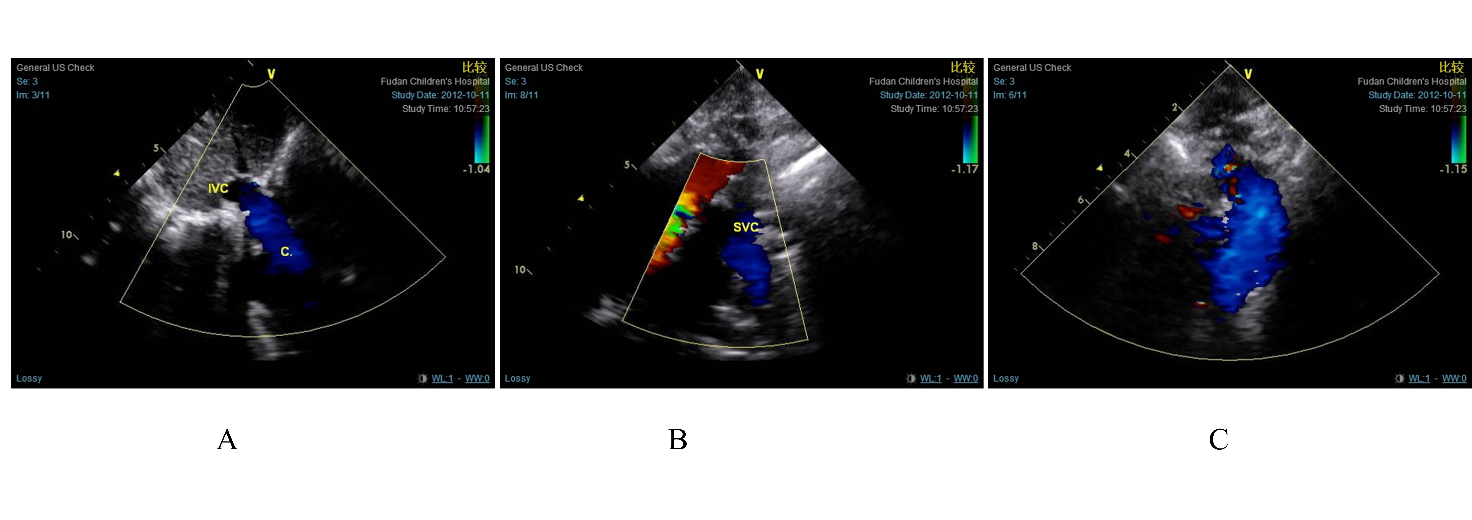


**Supplementary Figure 6.** Echocardiography Results for CH5078 carrying LOF variant (*DNA11*: c.12058_12059del: p.M4020fs)


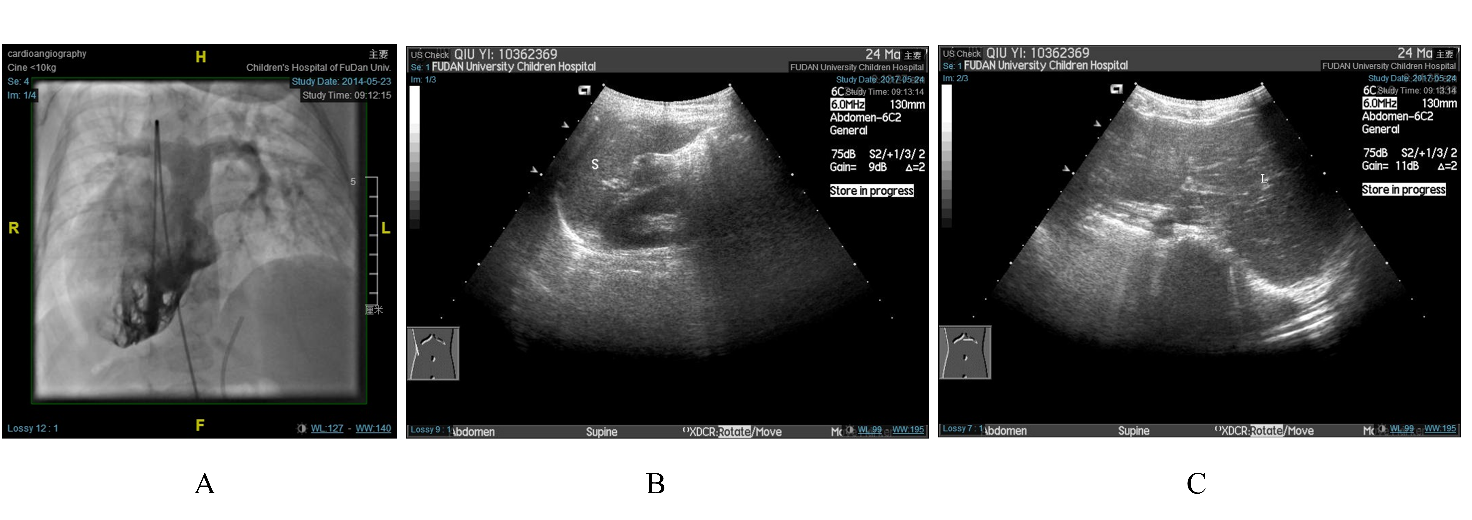


**Supplementary Figure 7.** Cardiac Catheterization and Abdominal Ultrasonography Results for CH5103 carrying LOF variant (*DNA11*: c.10214_10217del: p. K3405fs)

## Supplementary Tables

See Supplementary Table.xlsx

1. Li AH, Hanchard NA, Azamian M, D’Alessandro LCA, Coban-Akdemir Z, Lopez KN, et al. Genetic Architecture of Laterality Defects Revealed by Whole Exome Sequencing. *European Journal of Human Genetics* (2019) 27(4):563-73. doi: 10.1038/s41431-018-0307-z.

2. Jin SC, Homsy J, Zaidi S, Lu QS, Morton S, DePalma SR, et al. Contribution of Rare Inherited and De Novo Variants in 2,871 Congenital Heart Disease Probands. *Nat Genet* (2017) 49(11):1593-+. doi: 10.1038/ng.3970.

3. Ellesøe SG, Workman CT, Bouvagnet P, Loffredo CA, McBride KL, Hinton RB, et al. Familial Co-Occurrence of Congenital Heart Defects Follows Distinct Patterns. *European heart journal* (2018) 39(12):1015-22. doi: 10.1093/eurheartj/ehx314.

4. Zaidi S, Brueckner M. Genetics and Genomics of Congenital Heart Disease. *Circulation research* (2017) 120(6):923-40. Epub 2017/03/18. doi: 10.1161/CIRCRESAHA.116.309140.
